# Syncytiotrophoblast Extracellular Vesicles From Late-Onset Preeclampsia Placentae Suppress Pro-Inflammatory Immune Response in THP-1 Macrophages

**DOI:** 10.3389/fimmu.2021.676056

**Published:** 2021-06-07

**Authors:** Toluwalase Awoyemi, Carolina Motta-Mejia, Wei Zhang, Lubna Kouser, Kirsten White, Neva Kandzija, Fatimah S. Alhamlan, Adam P. Cribbs, Dionne Tannetta, Emily Mazey, Christopher Redman, Uday Kishore, Manu Vatish

**Affiliations:** ^1^ Nuffield Department of Women’s and Reproductive Health, University of Oxford, Oxford, United Kingdom; ^2^ Biosciences, College of Health, Medicine and Life Sciences, Brunel University London, Uxbridge, United Kingdom; ^3^ National Heart and Lung Institute, Imperial College London, London, United Kingdom; ^4^ Department of Infection and Immunity, King Faisal Specialist Hospital and Research Centre, Riyadh, Saudi Arabia; ^5^ Nuffield Department of Orthopaedics, Rheumatology and Musculoskeletal Sciences, University of Oxford, Oxford, United Kingdom; ^6^ Department of Food and Nutritional Sciences, University of Reading, Reading, United Kingdom

**Keywords:** preeclampsia, vesicles, inflammation, placenta, THP-1 cells

## Abstract

Syncytiotrophoblast derived Extracellular Vesicles (STBEV) from normal pregnancy (NP) have previously been shown to interact with circulating monocytes and B cells and induce pro-inflammatory cytokine release. Early-onset preeclampsia (EOPE) is associated with an exacerbated inflammatory response, yet there is little data regarding late-onset PE (LOPE) and immune function. Here, using a macrophage/monocyte cell line THP-1, we investigated the inflammatory potential of STBEV, comprising medium/large-STBEV (>200nm) and small-STBEV (<200nm), isolated from LOPE (n=6) and normal (NP) (n=6) placentae via dual-lobe *ex-vivo* placental perfusion and differential centrifugation. THP-1 cells bound and internalised STBEV isolated from NP and LOPE placentae, as revealed by flow cytometry, confocal microscopy, and ELISA. STBEV-treated THP-1 cells were examined for cytokine gene expression by RT-qPCR and the cell culture media examined for secreted cytokines/chemokines. As expected, NP medium/large-STBEV significantly upregulated the transcriptional expression of TNF-α, IL-10, IL-6, IL-12, IL-8 and TGF-β compared to PE medium/large-STBEV. However, there was no significant difference in the small STBEV population between the two groups, although in general, NP small STBEVs slightly upregulated the same cytokines. In contrast, LOPE STBEV (medium and large) did not induce pro-inflammatory responses by differentiated THP-1 macrophages. This decreased effect of LOPE STBEV was echoed in cytokine/chemokine release. Our results appear to suggest that STBEV from LOPE placentae do not have a major immune-modulatory effect on macrophages. In contrast, NP STBEV caused THP-1 cells to release pro-inflammatory cytokines. Thus, syncytiotrophoblast extracellular vesicles from LOPE dampen immune functions of THP-1 macrophages, suggesting an alternative mechanism leading to the pro-inflammatory environment observed in LOPE.

## Introduction

Preeclampsia (PE) is a pregnancy-specific syndrome, characterised by new-onset maternal hypertension and proteinuria or organ dysfunction, amongst other symptoms. PE is commonly divided into two subgroups: early-onset PE (EOPE), occurring ≤ 34-weeks gestation, and late-onset PE (LOPE), occurring ≥ 34-weeks gestation. The pathophysiology of EOPE is widely believed to be associated with the placenta (1). Immune imbalance (2–4) and cardiovascular dysfunction (5, 6) are highly significant in EOPE patients. The cause of the LOPE is more controversial, with some suggesting its placental origin (1, 7); another school of thought suggested maternal stress response as a pathogenic mechanism due to an incompatibility between the metabolic demands of the growing foetus close to term and maternal supply (8–10). The immunology of pregnancy involves crosstalk between a diverse population of immune and non-immune cells such as uterine natural killer cells (uterine NK cells), decidual macrophages, T cells, syncytiotrophoblast, decidual stromal cells and extravillous trophoblast cells through soluble factors like cytokines or chemokines and syncytiotrophoblast extracellular membrane vesicles (STBEVs). The uterine NK cells produce both pro (type 1 response) such as IL-12, IL-15, IL-18 and IFN-γ and anti-inflammatory cytokines (type 2 response) such as IL-4 and IL-10 (11), which interact with the human leukocyte antigen (HLA) antigens on the extravillous trophoblasts (EVT) (12). Similarly, T cells, in particular T helper cells, mainly exist in the form of Th1 or Th2 phenotype. The Th1 phenotype predominates (5-30% of T cells) (13) in the first trimester produce pro-inflammatory cytokines such as IL-2, TNF-α and IFN-γ (type 1 response), recruit essential immune cells and help direct implantation, regulate trophoblast invasion by altering trophoblast cell adhesion to laminin (14), elevate plasminogen activator inhibitor-1 (15, 16), and prepare for the labour (17). IFN-γ regulates peri-implantation vascular remodelling (18, 19) and extra villous trophoblast invasion. Th2 cells release type 2 cytokines such as IL-10, IL-4 and IL-13 and are critical in immunosuppressing the pro-inflammatory responses. Like T helper cells, decidual macrophages predominate in two forms: M1 which are involved in the type 1 pro-inflammatory response, and M2 macrophages which are involved in anti-inflammatory type 2 response. Like Th1 and Th2, M1 and M2 are antagonistic in their downstream functions and co-regulate each other’s actions but may switch depending on the microenvironment between phenotypes.

There is a failure of this immunotolerance in PE, resulting in poor placentation. However, no clear immunological distinction has been made between EOPE and LOPE. Elevation of type 1/pro-inflammatory and reduction of type 2/anti-inflammatory cytokines have been detected in the serum and placenta of PE patients (20). In addition, epidemiological studies that have found an association between PE and primi-paternity (21, 22), and sperm exposure (23) highlight a potential role of immunology in the pathogenesis of PE. Two types of Th1/Th2 predominant type, PE have been described, a more common Th1 predominant type and a less common Th2 predominant type. In the Th2 predominant type, PE is caused by the production of allo-or autoantibodies (antiphospholipid antibodies), which can inhibit the invasion of trophoblasts in the decidua (11). This has also been observed in macrophages with a polarisation to M1 phenotype observed in PE (24). In addition, studies have identified an increased number and activation of macrophage in PE (21, 22), which may contribute to the limited trophoblast invasion and spiral artery remodelling.

Extracellular vesicles (EV) are membrane-bound complexes extruded from cells and are potent signalling modulators of the immune system under both physiological and pathological conditions (25). In pregnancy, the syncytiotrophoblast (STB) and the maternal immune system communicate *via* the release of soluble factors such as chemokines, cytokines, complement, steroid and protein hormones, as well as by factors carried by EV (26). As recommended by the MISEV 2018 guidelines (27), STB derived EVs (STBEV) comprise two subgroups according to their size: medium/large STBEV (> 200 nm) and small STBEV (< 200 nm) (28). STBEVs have immunomodulatory roles during pregnancy (26, 28, 29). STBEVs, isolated from perfused normal placentae (NP), have been shown to bind to B cells and monocytes, and enhance the release of pro-inflammatory cytokines by peripheral blood mononuclear cells (PBMCs) and monocytes (30–33). Medium/large STBEVs isolated from PE placental explants have also been shown to further enhance the secretion of pro-inflammatory cytokines and chemokines by PBMCs, including IL-1β, when compared to NP medium/large STBEV (34). Similarly, PBMCs, treated with medium/large STBEV derived from trophoblast cells grown under hypoxic conditions release higher IL-6 and TNF-α compared to PBMCs cultured alone (35).

STBEVs have been shown to circulate in increased levels in maternal peripheral plasma (36), especially in EOPE (37, 38). The different STBEV subtypes (medium/large and small STBEV) and their interaction with macrophages have not been studied. Previous studies have (a) used pooled EVs (which do not distinguish the effects of small STBEVs from medium/large STBEVs); (b) used term placentae taken from a caesarean section or vaginal delivery [vaginal delivery subjects placenta to labour stresses (39)]; or (c) not clearly defined the PE disease state (30, 31, 33). Taking into account that research has been mostly focused on EOPE placentae, we decided to concentrate our studies on LOPE derived STBEVs.

Our hypothesis was that STBEVs derived from LOPE placentae would cause an exacerbated inflammatory effect on human THP-1 macrophages compared to STBEV obtained from the placenta from normal pregnancy similar to that seen from EOPE. To investigate this, we examined (1) whether THP-1 cells would internalise STBEV derived from NP or LOPE perfused placentae, (2) if there was any difference in the uptake of STBEV derived from NP and LOPE placentae; and (3) whether STBEV isolated from LOPE placentae would induce an altered inflammatory response to THP-1 compared to those derived from NP.

## Materials and Methods

### Human Subjects

The human subjects’ material used in this project was approved by the Central Oxfordshire Research Ethics Committee C (REF 07/H0607/74 & 07/H0606/148). All mothers undergoing elective caesarean section without labour were consented for the use of their placentae by research midwives. Mothers suffering from PE were recruited using the corresponding diagnostic criteria defined by the International Society for the Study of Hypertension in Pregnancy (ISSHP) (40). According to ISSHP, PE is defined as *de novo* hypertension in the second half of pregnancy (>140/90 mm Hg) accompanied with one or more complications: new proteinuria (>300mg per 24 hours), or another maternal organ dysfunction. The clinical characteristics of PE patients and controls (NP) are summarised in **Table 1**.

**Table 1:** Clinical data of human subjects.

	Placental STBEV		P value
	NP (n=6)	PE (n=6)	
**Age (years)**	37.3 ± 0.7	32.7 ± 4.7	ns
**Gestation Age (weeks + days)**	38+1 ± 6.5	36+0 ± 5	*
**Mean no. of pregnancies**	2.3 ± 0.3	0.7 ± 0.3	**
**Body Mass Index (kg/m^2^)**	27.3 ± 2.4	37.1 ± 6.7	ns
**Max. Proteinuria qPCR (mg/mmol)**	16.1 ± 8.6	256.7 ± 168.7	**
**Max. Systolic pressure (mm Hg)**	137.3 ± 1.8	181 ± 15.9	**
**Max. Diastolic pressure (mm Hg)**	81.7 ± 8.7	108.7 ± 6.3	**
**New-born weight (g)**	4008 ± 137.2	2418 ± 66	**
**Smoking History**	3/6	2/6	ns

Data presented as Mean ± SEM, significant difference shown as p < 0.05 (*), p < 0.01 (**) or ns, not significant.

### Isolation and Characterisation of STBEV

STBEVs were prepared using a modified dual-lobe placental perfusion system with human placentae, following differential centrifugation, and characterised, as previously described (41). Briefly, a suitable placenta lobe without calcifications or area of necrosis was identified, cannulated, and perfused with Medium 199 with L-glutamine and Earle’s salts containing 0.5% w/v Bovine serum albumin (BSA; Sigma U.K), 0.8% Dextran 20 and 500 U/L sodium heparin with (foetal) or without (maternal) dextran for 3 h. The resultant maternal perfusate was initially centrifuged at 2500 x g twice to get rid of red blood cells and cell debris. The supernatant was then centrifuged at 10,000 x g for 30 minutes at 4°C, the pellet was resuspended in filtered PBS and termed as medium/large STBEVs; the supernatant was further centrifuged at 150,000 x g for 2 h at 4°C and the pellet resuspended in filtered PBS and classified as small STBEVs. Leukocyte depletion was done with the human CD45 depletion kit (EasySep™). The two fractions were characterised by transmission electron microscopy (TEM), standard flow cytometry, nanoparticle tracking analysis (NTA), and western blot, which confirmed that our fractions were indeed enriched for medium/large and small STBEVs, respectively.

### Cell Culture

THP-1 cells, originally derived from human acute monocyte leukaemia cell line (ATCC^®^ TIB-202™), were cultured in RMPI-1640 medium (Gibco) containing 10% v/v fetal calf serum (FCS), 2mM L-glutamine, 100U/mL penicillin, and 100 µg/mL streptomycin. FCS was centrifuged at 150,000 x g for 18 h using a Beckman L8-80M ultracentrifuge and filtered at 0.1 μm under sterile conditions to reduce possible contamination of FCS EVs. THP-1 cells were adjusted to 5 x 10^5^ cells per well in a 24-well plate and differentiated into macrophage-like phenotype using 50 ng/mL of Phorbol 12-Myristate 13-Acetate (PMA; Sigma) in RPMI-1640 complete medium (including 10% v/v EV-free FCS) for 48 h at 37°C under 5% v/v CO_2_.

### THP-1 Treatment With STBEV

Medium/large STBEV from 6 NP were pooled together based on equal protein concentration. This was also true for NP small STBEV, LOPE medium/large STBEV and LOPE small STBEV. It has been widely reported that there is a greater shedding/release of STBEV in PE, especially in EOPE (36). Furthermore, it is likely that different sized EVs will express different protein cargo based on their heterogeneity. Thus, we chose to use the same particle number rather than protein concentration. To do this, medium/large STBEV and small STBEV were analysed by Nanoparticle Tracking Analysis (NTA); further dilution in serum-free media was carried out to achieve the same particle number among NP and PE medium/large STBEV or small STBEV (1 x 10^9^ particles/mL). STBEVs were incubated with differentiated THP-1 cells for 2 and 6 h for qPCR, and 12 and/or 24 h for multiplex cytokine/chemokine array analysis. THP-1 cells with no STBEV were used as a control for each time point. The supernatant was collected, and dead cells and debris were removed by centrifugation at 1,500 x g for 10 minutes at 4°C, and then stored at -80°C.

### Flow Cytometry Analysis of THP-1 Cells Treated With Bio-Maleimide Stained STBEV

Medium/large STBEV from NP or LOPE placentae were pre-stained with 2 µM of Bio-maleimide (Molecular Probes) [previously filtered using a 0.2 µm nanosep centrifugal device (VWR)] for 30 minutes at room temperature in the dark and washed with PBS by centrifugation at 10,000 x g at 4°C for 35 minutes prior to flow cytometry analysis. After treatment of STBEV (1 x 10^9^ particles/mL) to THP-1 macrophages (5 x 10^5^ cells/well), cells were lifted with Trypsin-EDTA (Sigma) and fixed with 1 mL of 2% v/v paraformaldehyde (PFA). Finally, the pellet was re-suspended in 300 µL of filtered PBS and 5,000 events were recorded per sample using FACS Diva software (BD Biosciences).

### Confocal Microscopy of THP-1 Cells Treated With PKH26-Stained STBEV

STBEVs from each patient group were pre-stained with PKH26 dye (red; MINI26; Sigma), the THP-1 cells’ membrane were labelled with WGA-Alexa 488 (green; Vector Laboratories), and the cell nuclei with Hoechst 33342 (blue; Sigma). THP-1 cells alone were used as a control. Cells were viewed under a Zeiss confocal microscope with a Yokogawa spinning disk scanning unit and an attached Evolve^®^ 512 Delta EMCCD camera, and images were taken using Zen Blue software (Zeiss). Z-stacks (11 slices) were taken through a 10µm depth, and pictures were compressed on maximum intensity using ImageJ software (Fiji).

### PLAP ELISA

Microtitre wells in a 96-well MaxiSorp plate (Nunc) were coated overnight with 100 µL of 10 µg/mL PLAP antibody (NDOG2). The wells were washed with PBS-T (PBS + 0.05% Tween 20) and incubated with 300 µL of blocking buffer (5% w/v BSA in PBS-T) for 3 hours. For ELISA, NP medium/large STBEVs derived from 3 mothers were pooled and serially diluted (4,000 ng/mL down to 1 ng/mL) in 1% BSA + PBS-T. Untreated and treated THP-1 cells were extensively washed to get rid of uninternalised and unbond STBEVs, after which both groups of THP-1 cells were added to the plate and incubated in triplicates. Following overnight incubation, the wells were thoroughly washed using PBS-T to get rid of unbound targets. 1-Step pNPP (*p*-nitrophenyl phosphate disodium salt) substrate kit (Sigma) was used to detect alkaline phosphatase activity. 100 µL of the substrate was added to each well and incubated for 1 h and 30 minutes. 50 µL of 2N sodium hydroxide (NaOH) was added to stop the reaction. Absorbance was measured at 405nm using the FluoStar OPTIMA (BMG) plate reader.

### Real-Time Quantitative PCR for Cytokines Expression

RNA was extracted using the RNeasy micro kit (Qiagen), followed by treatment with DNA-free removal kit (ThermoFisher) to remove any contaminating genomic DNA prior to RT-qPCR reaction. The RNA concentration was measured using a NanoDrop ND-1000 Spectrophotometer (ThermoFisher) at 260 nm absorbance, and RNA purity was assessed using the 260:280 nm absorbance ratio. Samples were diluted with RNase-free water and adjusted to 100 ng/μL concentration. RNA samples were converted into cDNA using a High capacity RNA-cDNA conversion kit (Applied Biosystems).

The nucleotide Basic Local Alignment Search Tool and Primer-BLAST were used to design and analyse the specificity of the primer sequences. Primers sequences are listed in **Table 2**.

**Table 2:** Primers sequences used for real-time quantitative polymerase chain reaction (qPCR).

Gene Targets	Forward Primer	Reverse Primer	Source
**18S**	5’-ATGGCCGTTCTTAGTTGGTG-3’	5’-CGCTGAGCCAGTCAGTGTAG-3’	Sigma-Aldrich
**TNF-α**	5’-AGCCCATGTTGTAGCAAACC-3’	5’-TGAGGTACAGGCCCTCTGAT-3’	Sigma-Aldrich
**TGF-β**	5’-GTACCTGAACCCGTGTTGCT-3’	5’-GTATCGCCAGGAATTGTTGC-3’	Sigma-Aldrich
**IL-10**	5’-TTACCTGGAGGAGGTGATGC-3’	5’-GGCCTTGCTCTTGTTTTCAC-3’	Sigma-Aldrich
**IL-12**	5’-AACTTGCAGCTGAAGCCATT-3’	5’-GACCTGAACGCAGAATGTCA-3’	Sigma-Aldrich
**IL-8**	5’-CTGTGTGAAGGTGCAGTTTTG-3’	5’-GTGTTGGCGCAGTGTGGTC-3’	Sigma-Aldrich
**IL-6**	5’-GAAAGCAGCAAAGAGGCACT-3’	5’-TTTCACCAGGCAAGTCTCCT-3’	Sigma-Aldrich

RT-qPCR reaction contained 3.7 μL of RNase-free water, 5 μL Power SYBR Green Master mix (Applied Biosystems), 75 nM of forward and reverse primers and 100 ng template cDNA in a final reaction volume of 10 μL, run in a 7900HT fast Real-Time RT-qPCR system (Applied Biosystems). The RT-qPCR reaction initially included 2 minutes of incubation at 50°C, followed by 10 minutes incubation at 95°C. The template was then amplified for 40 cycles under the following conditions: 15 seconds incubation at 95°C and 1-minute incubation at 60°C. The single housekeeping gene, 18S, was included in each RT-qPCR reaction set to normalise the rest of the samples against the expression of human 18S rRNA. Data were acquired using the RQ Manager Version 1.2.1 (Applied Biosystems). Ct (cycle threshold) values for each target gene expression were calculated, and Relative Quantification (RQ) values for each cytokine target gene was calculated using the formula: RQ= 2^-ΔΔCt^. Assays were carried out twice in triplicate.

### Multiplex Cytokine Array Analysis

Supernatant samples collected in previous THP-1-STBEV treatment experiments were analysed using a Milliplex ^®^ MAP Human cytokine/chemokine magnetic bead panel kit (HCYTOMAG-60K; EMD Millipore). Briefly, in a 96-well plate, 25 μL of assay buffer was added to each well. This was followed by the addition of 25 μL of standard, control and supernatant samples. The 96-well plate was washed with assay buffer, and 25 μL of target antibodies were incubated with the beads for 1 h at room temperature. 25 μL of Streptavidin-Phycoerythrin conjugate was then added to each well and incubated for 30 minutes in the dark at room temperature. After washing step with the assay buffer, 150 μL of sheath fluid was added to each well, and the plate was read using the Luminex Magpix instrument.

### Statistical Analysis

The data were analysed using GraphPad 9 software. Multiplex cytokine array data were analysed with one-way ANOVA and presented as mean ± SEM cytokine concentration. Multiple hypothesis testing was corrected for using statistical hypothesis testing (Tukey). For qPCR analysis, one-way ANOVA was done on the ΔCT values with a similar statistical method applied as for the multiplex cytokine array data analysis. Graph for the qPCR data was presented as mean ± SEM log(2)fold change.

## Results

### Characterisation of Small and Medium/Large STBEVs by Western Blot (WB), Nanoparticle Tracking Analysis (NTA), Flow Cytometry, and Transmission Electron Microscopy (TEM)

**Figure 1:**
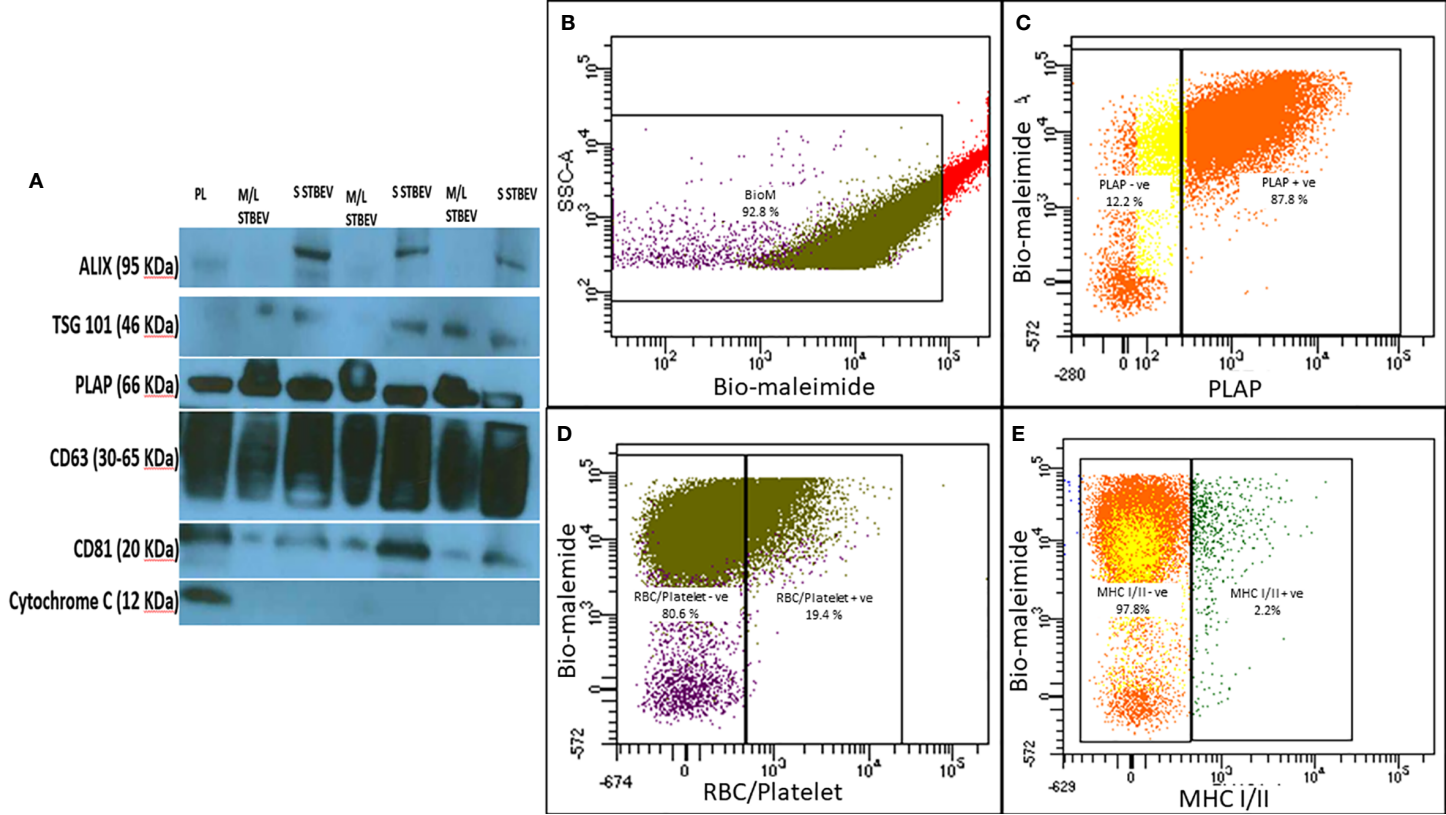
**(A)** Representative Western blot image of placenta lysate (PL), medium/large STBEVs (n=3) and small STBEVs (n=3) showing expression of extracellular vesicle markers (ALIX, TSG101, CD63 and CD81), syncytiotrophoblast origin marker (PLAP) and negative expression of cytochrome **(C)**. **(B–E)** are representative flow cytometry analysis graphs of medium/large STBEVs derived from the placentae. **(B)** the per cent of vesicles in the medium/large STBEVs enriched sample fraction (based on the per cent of bio-maleimide positive events), **(C)** the per cent of PLAP positive extracellular vesicles, **(D)** the per cent of MHC I/II negative extracellular vesicles. In contrast, **(E)** shows the per cent of platelet and endothelial cells derived vesicles in our samples.

**Figure 2:**
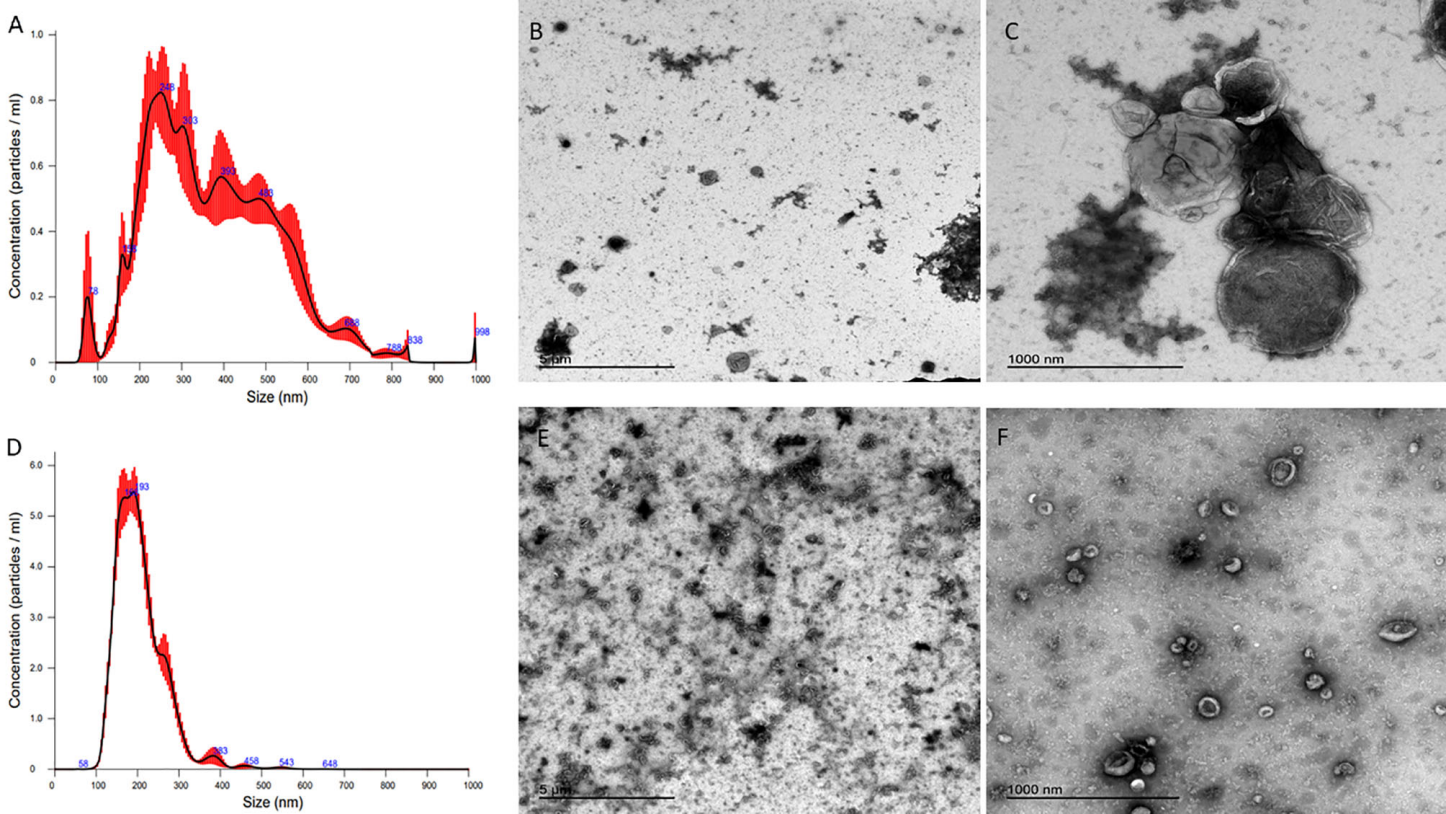
**(A, D)** are nanoparticle tracking analysis (NTA) graphs of medium/large and small STBEVs, respectively, while **(B)** (wide angle) and **(C)** (close-up) are transmission electron microscopy images of medium/large STBEVs. **(E)** (wide angle) and **(F)** (close up) are transmission electron microscopy images of small STBEVs.

Characterisation of enriched STBEV fractions was confirmed using western blotting assays for the classical EV markers, tetraspanins (CD81, CD63), an endosomal trafficking protein marker (ALIX and TSG101) and a non-EV marker (Cytochrome C) as negative control (**Figure 1A**). Immunoblotting revealed the presence of placenta alkaline phosphatase (PLAP; 66 KDa), typical EV markers [CD63 (30–65 KDa), CD81 (20 KDa), and ALIX (95 KDa) and TSG101 (46 KDa)] (**Figure 1**), while the cytochrome C (12 KDa), the negative EV marker was not detected in the STBEV fractions but was detected in the placenta lysate. TSG101 and ALIX were more prominent in the small STBEV fractions than the medium/large STBEV fractions and placenta lysate. Flow cytometric analysis showed that most of the particles analysed were vesicular (greater than 240 nm and less than 1µm in size and 92.8% Biomalemide positive (**Figure 1B**) and syncytiotrophoblast derived (87.8% express PLAP; **Figure 1C**, 97.8% are MHC class I/II negative; **Figure 1E**) with little contamination from platelets and red blood cell vesicles (only 19.4% of the vesicles are platelets and red blood cell-derived; **Figures 1C, D**). We could not study the small STBEVs because they were below the level of detection of a standard flow cytometer. Nanoparticle tracking analysis for the size distribution of medium/large and small STBEV fractions showed a modal size of 245.8 ± 7.9 nm and 176.3 ± 7.5 nm, respectively (**Figures 2A, D**). A close-up view of transmission electron microscopy (TEM) revealed the characteristic ‘cup shaped’ morphology and size range of extracellular vesicles in both medium/large (**Figure 2C**) and small (**Figure 2F**) STBEV fractions. **Figures 2B, E** show wide-angle view of medium/large and small STBEV fractions, respectively, along with their co-isolates. Together, these results confirmed that our fractions were enriched for small and medium/large STBEVs.

### Medium/Large STBEV From NP and LOPE Are Taken Up by Differentiated THP-1 Cells

**Figure 3:**
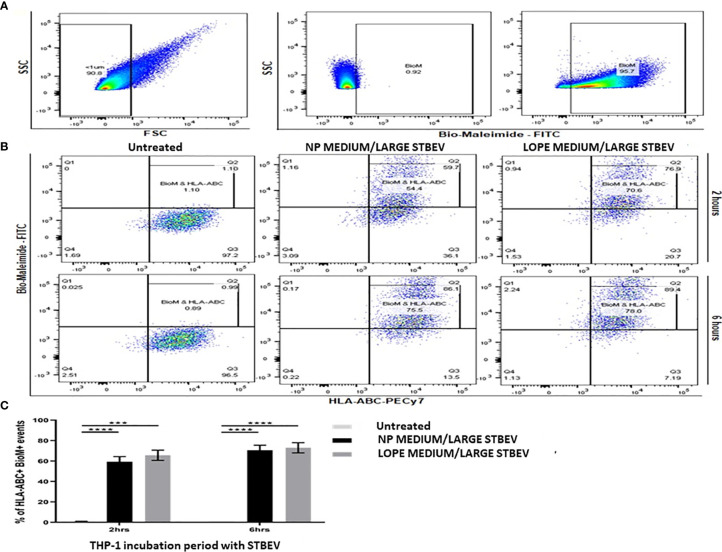
Flow cytometry analysis of the uptake of medium/large STBEV derived from NP and PE placentae by differentiated THP-1 cells (n=3). Bio-Maleimide pre-stained medium/large STBEV characterisation **(A)**. Representative SSC vs FSC graph with chosen EV population to be less than 1 micron in size (characterised as microvesicles), followed by SSC vs Bio-Maleimide-FITC channel graph showing the chosen 1% cut-off gate using non-stained medium/large STBEV and Bio-Maleimide stained medium/large STBEV. Treatment of Bio-Maleimide stained medium/large STBEV to HLA-ABC stained THP-1 macrophages **(B)**. Representative Bio-Maleimide-FITC vs HLA-ABC-PeCy7 graph at 2 and 6 h treatment showing untreated THP-1, then treated ThP-1 with medium/large STBEV derived from NP and PE placentae. Bar-graph demonstrating merged results from THP-1 macrophages untreated and treated with NP and PE medium/large STBEV **(C)** plotted as HLA-ABC^+^ Bio-Maleimide^+^ against incubation period. Data presented as Mean ± SEM, significant difference shown as p<0.001 (***) and p<0.0001 (****).

Uptake of NP and LOPE medium/large STBEV by differentiated THP-1 cells were investigated by flow cytometry *via* Bio-maleimide (STBEV membrane marker, FITC) and HLA-ABC (THP-1 cell marker; PeCy7) co-expression (n=3, **Figure 3**). Our data revealed that treatment of THP-1 cells with NP and LOPE medium/large STBEV showed a double positivity (FITC^+^PeCy7^+^) at 2 h (NP: 70% ± 5.6, LOPE: 54% ± 8.6; p>0.05) and 6 h (NP: 75% ± 4.2, LOPE: 78% ± 6.6; p>0.05), demonstrating uptake of medium/large STBEV by THP-1 cells (Quadrant Q2-2; **Figure 3A**). Although the uptake of medium/large STBEV in THP-1 cells (**Figures 3B, C**) revealed a significant difference in the co-expression of Bio-maleimide and HLA-ABC compared to untreated cells (2 h: p ≤ 0.01; 6 h: p ≤ 0.001; **Figure 3C**), there was not a significant difference in uptake between medium/large STBEV derived from NP and LOPE placentae (p= 0.021; **Figure 3C**), suggesting a comparable level of phagocytosis. We could not study the small STBEVs because they were below the level of detection of standard flow cytometer.

### Confocal Microscopy Confirmed Internalisation of STBEV From NP and LOPE Placentae Into THP-1 Macrophages

**Figure 4:**
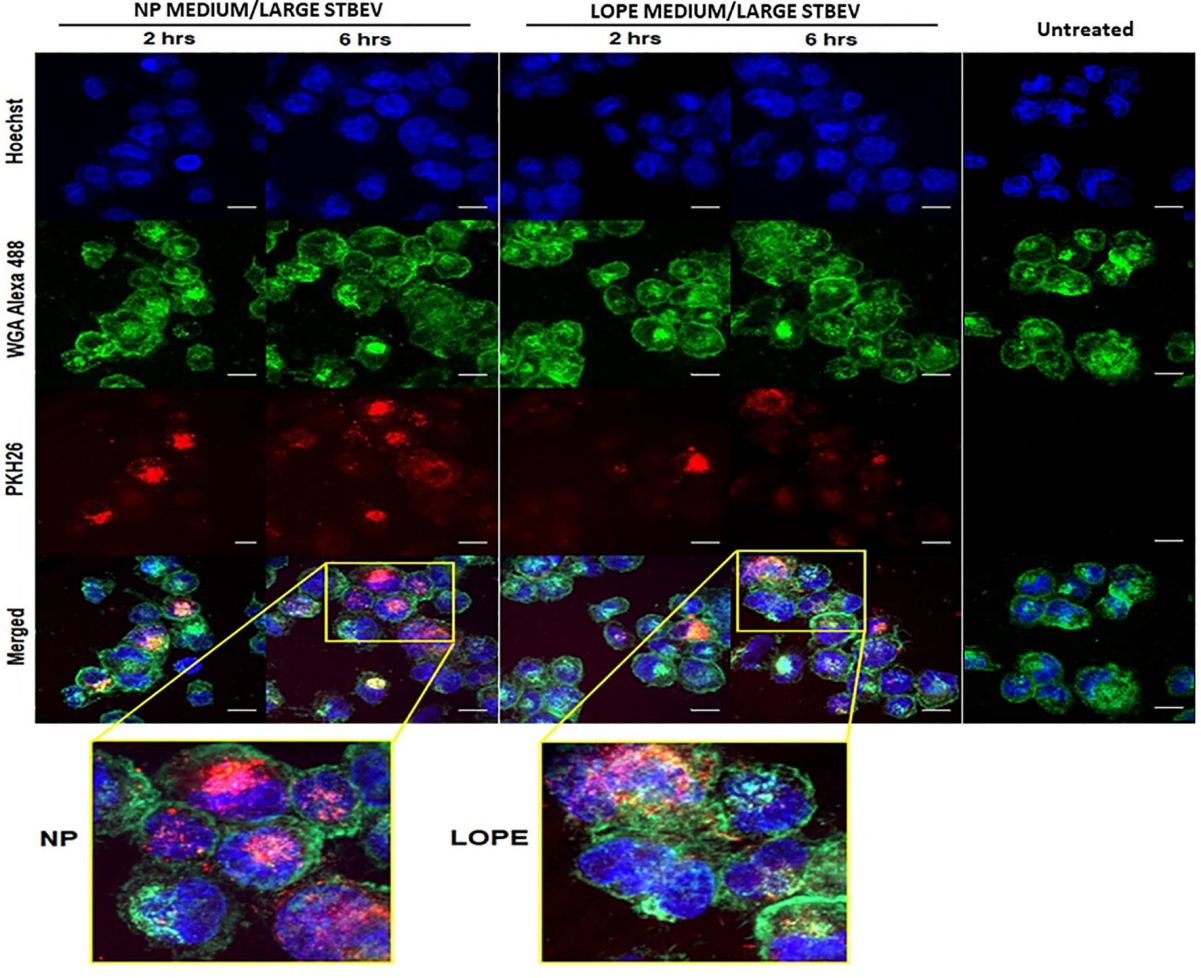
The internalisation of medium/large STBEVs from NP and PE placentae into THP-1 cells by confocal microscopy (technical replicate =3). Medium/large STBEVs derived from NP and PE patients incubated for 2 and 6 hours incubation with differentiated THP-1 cells. Channels panel show cells’ nuclei labelled with Hoechst dye (blue), cells’ membrane labelled with WGA Alexa 488 (green), STBEVs labelled with PKH26 dye (red) and merged channels. ThP-1 only incubated with no STBEVs are used as the control. This control also served as the control for the experiment to detect internalisation of small STBEVs from NP and PE placentae into THP-1 cells (**Figure 5**). Scale bars, 10 μm.

**Figure 5:**
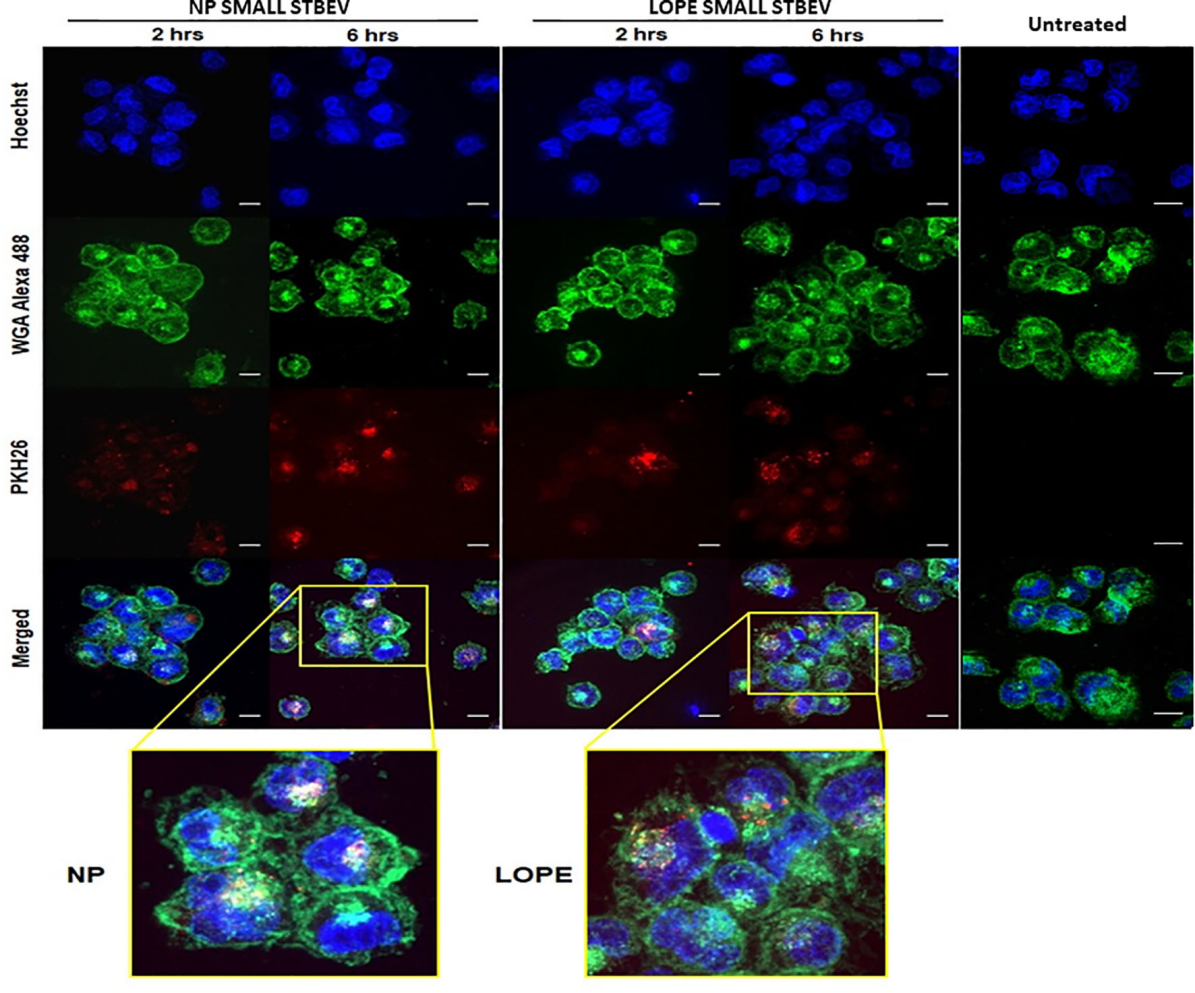
The internalisation of small STBEVs from NP and PE placentae into THP-1 cells by confocal microscopy (technical replicate =3). Small STBEVs derived from NP and PE patients incubated for 2 and 6 hours incubation with differentiated THP-1 cells. Channels panel show cells’ nuclei labelled with Hoechst dye (blue), cells’ membrane labelled with WGA Alexa 488 (green), STBEVs labelled with PKH26 dye (red) and merged channels. THP-1 only incubated with no STBEVs (from **Figure 4**) was used as the control. Scale bars, 10 μm.

Confocal microscopy was used to assess whether STBEV (technical replicate = 3) were being internalised by differentiated THP-1 cells at different time points (2 and 6 h; **Figures 4** and **5**). Medium/large STBEV, derived from NP and LOPE medium/large STBEV placentae (**Figure 4**) and NP and LOPE small STBEV (**Figure 5**), appeared to be internalised by THP-1 cells. The internalisation of STBEV by THP-1 cells appear to be time-dependent as the images obtained after six hours looked more intensely stained than those obtained after two hours of incubation for both LOPE and NP.

### PLAP ELISA Confirmed Internalisation of STBEV From NP and LOPE Patients Into THP-1 Macrophages

**Figure 6:**
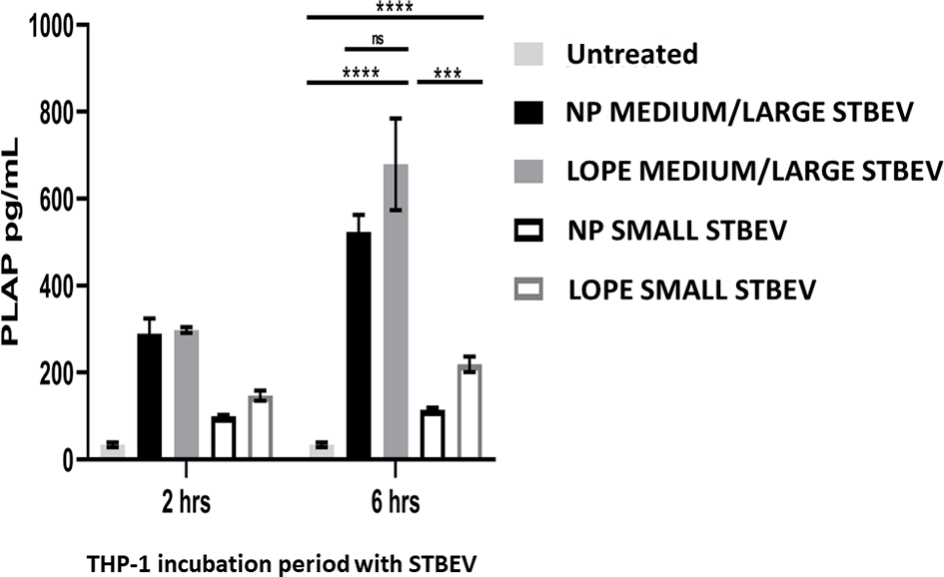
ELISA using STB specific marker, PLAP, on THP-1 macrophages treated with STBEVs derived from NP and PE placentae at 2 and 6 hours incubation. THP-1 cells do not innately express PLAP while STBEVs do. The presence of detectable PLAP in treated THP-1 cells indicate internalisation or/and fusion. Quantification analysis of NP and PE medium/large STBEVs and small STBEVs expressing PLAP marker internalised by THP-1 macrophages. Data presented as Mean ± SEM, significant difference shown as p<0.001 (***) and p<0.0001 (****). ns, non-significant.

To quantitate the STBEV taken up by THP-1 cells, PLAP (a specific marker of STBEV, thus not natively expressed in THP-1 cells) was measured using a standard ELISA. We confirmed that STBEV were indeed taken up by THP-1 cells (n=3; **Figure 6**). This quantification included the internalised STBEV and those bound to the cell membrane of THP-1. Medium/large STBEV uptake by THP-1 was also time-dependent, although there was no significant difference between each group, i.e., NP and LOPE (small STBEV uptake showed a significant difference between NP and LOPE at 6-h time-point (NP < LOPE small STBEV: p<0.001). These findings corroborate our confocal microscopy data in regard to small STBEV internalisation by THP-1 cells. However, we should take into account that PLAP expression is reduced in PE derived STBEV (42). Hence, we can only determine that both medium/large STBEV and small STBEV from NP and LOPE are taken up by THP-1 without a significant difference.

### NP Medium/Large STBEV Significantly Upregulated mRNA Expression of Pro-Iinflammatory Cytokines in THP-1 Macrophages

**Figure 7:**
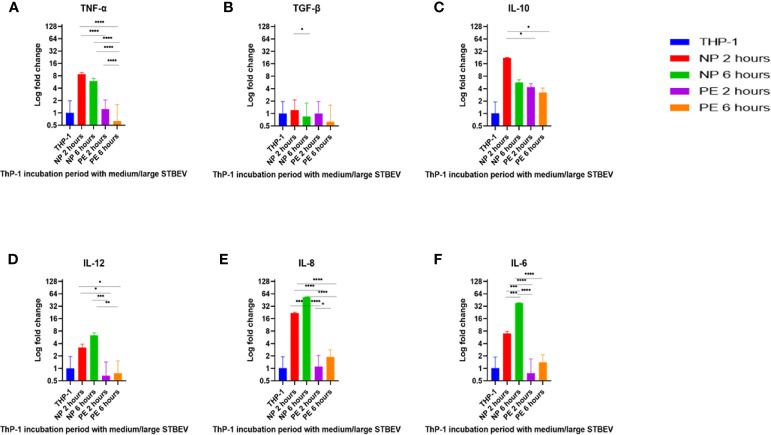
RT-qPCR analysis of the medium/large STBEV induced expression of cytokines by differentiated ThP-1 cells. Cells were incubated with medium/large STBEVs from NP and PE placentae at 2 and 6 hour time-points. In control experiments, cells were incubated with filtered PBS at the same time-points. mRNA expression was measured using real-time RT-qPCR for medium/large STBEVs; TNF-α **(A)**, TGF-β **(B)**, IL-10 **(C)**, IL-12 **(D)**, IL-8 **(E)** and IL-6 **(F)**. Data were normalised to 18S rRNA expression that was used as an internal reference gene. Values calculated as mean ± SEM of value from cells treated with 1 X 10^9^ of medium/large STBEVs. To determine significant difference in expression, one-way ANOVA was performed on data: *p ≤ 0.05, **p ≤ 0.01, ***p ≤ 0.001 and ****p < 0.0001.

**Figure 8:**
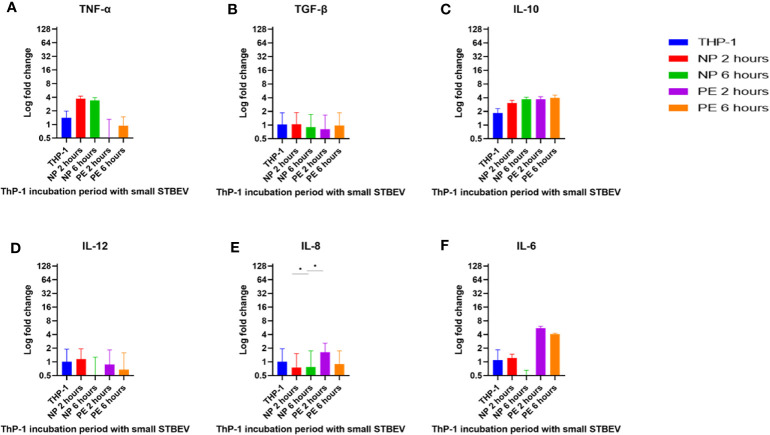
RT-qPCR analysis of the small STBEV induced expression of cytokines by differentiated THP-1 cells. Cells were incubated with small STBEVs from NP and PE placentae at 2 and 6 hour time-points. In control experiments, cells were incubated with filtered PBS at the same time-points. mRNA expression was measured using real-time RT-qPCR for small STBEVs; TNF-α **(A)**, TGF-β **(B)**, IL-10 **(C)**, IL-12 **(D)**, IL-8 **(E)** and IL-6 **(F)**. Data were normalised to 18S rRNA expression that was used as an internal reference gene. Values calculated as mean ± SEM of value from cells treated with 1 X 109 of STBEV. To determine significant difference in expression, one-way ANOVA was performed on data: *p ≤ 0.05.

RT-qPCR analysis was used to investigate the gene expression of a selected group of cytokines in differentiated THP-1 cells incubated with STBEV derived from NP and LOPE placentae (n=3; **Figures 7** and **8**) for 2 and 6 h. Analysis was done with the ΔΔ CT (43) method, and statistical testing was done on the ΔCT values.

At 2 h of incubation, TNF-α mRNA was significantly upregulated by NP medium/large STBEV (fold change of 8.735 relative to untreated THP-1 cells hereafter referred to as FC, **Figure 7A**), compared to PE medium/large STBEV (FC of 1.24) while TNF-α mRNA was upregulated by NP small STBEV (FC of 3.72) compared to LOPE small STBEV (FC of 0.37, **Figure 8A**) though this difference was not significant. After 6 h of incubation, TNF-α was upregulated by NP medium/large STBEV (FC of 5.95) in comparison to LOPE medium/large STBEV (FC of 0.63) (medium/large STBEV: at 2 h p< 0.0001, at 6 h p< 0.0001) while no significant difference was found in small STBEV, but the NP group appeared to mildly increase transcript level while the LOPE group had suppressed transcript levels.

Similarly, at 2 h, IL-6 was highly expressed in THP-1 cells incubated with NP medium/large STBEV (FC of 7.06, **Figure 7F**), and its expression increased significantly over time (FC of 38.21). NP medium/large STBEV brought about a greater change in IL-6 expression compared to LOPE medium/large STBEV at both 2 and 6 h of incubation (p< 0.0001). There was no significant difference in the induction of IL-6 in NP compared to LOPE small STBEV (**Figure 8F**).

Likewise, IL-12 was more expressed at 2 h in cells treated with NP medium/large (**Figure 7D**) STBEV compared to LOPE (NP FC= 3.21 LOPE FC= 0.66, P < 0.01) and at 6 h (NP FC= 6.39 LOPE FC= 0.77, P < 0.01). There was no significant difference in cells treated with small STBEV from both groups. However, unlike NP SMALL STBEV at 2 h, which caused a modest upregulation in IL-12, the rest seem to have reduced expression compared to untreated THP-1 cells [(NP FC (2 h) = 1.15, LOPE FC (2 h)= 0.88, NP FC (6 h) = 0.52 LOPE FC (6 h)= 0.678, **Figure 8D**)].

There was no significant difference in TGF-β expression between small (**Figure 8B**) and medium/large STBEV (**Figure 7B**) between NP and LOPE; both groups did not cause an increased expression of TGF- β compared to untreated THP-1 cells. However, at 2 h of incubation, IL-10 mRNA was significantly upregulated by NP medium/large STBEV (FC of 22.23) compared to LOPE medium/large STBEV (FC of 4.36, p< 0.0001), but at 6 h, there was no significant difference between both groups (**Figure 7C**). Whilst treatment of THP-1 cells with small STBEV resulted in an upregulation of the transcript [FC: PE small STBEV (2 h)-3.70, NP small STBEV (2 h)-3.06, PE small STBEV(6 h)-3.95, NP small STBEV(6 h)-3.69, **Figure 4I**], this was not significantly different between NP and LOPE.

Besides, LOPE medium/large STBEV significantly increased expression of IL-8 compared to NP at both 2 and 6 h (p< 0.0001; **Figure 7E**). There was a slight suppression of IL-8 with small STBEV from NP at 2 h (FC=0.75), which increased at 6 h (FC=1.63, **Figure 8E**) while LOPE small STBEV failed to induce IL-8 expression. However, there was no significant difference between the two groups at both 2 and 6 h.

Overall, THP-1 macrophages challenged with medium/large NP STBEV showed upregulation of pro-inflammatory cytokines. Although small STBEV did not induce significant alterations in the cytokine profile consistent with the higher uptake, they still modestly increased the levels of transcripts of pro-inflammatory cytokines. Surprisingly, LOPE STBEV brought about modest changes in the expression of pro-inflammatory cytokines whilst LOPE small STBEV induced upregulation of IL-10 transcripts and downregulated the expression of other transcripts at 2 h. These results appear to suggest that NP-derived medium/large STBEV and small STBEV induce pro-inflammatory responses, whereas those derived from LOPE placentae (whilst clearly being internalised) do not cause an inflammatory response. The finding of upregulated IL-10 by LOPE derived small STBEV appears to suggest an anti-inflammatory and repair effect.

### NP-STBEV Cause a Significant Release of TNF-α, IL-8, IL-6, MIP1-α IP-10, MCP-1, VEGF, IL-1β, and GM-CSF From THP-1 Cells Compared to LOPE STBEV

**Figure 9:**
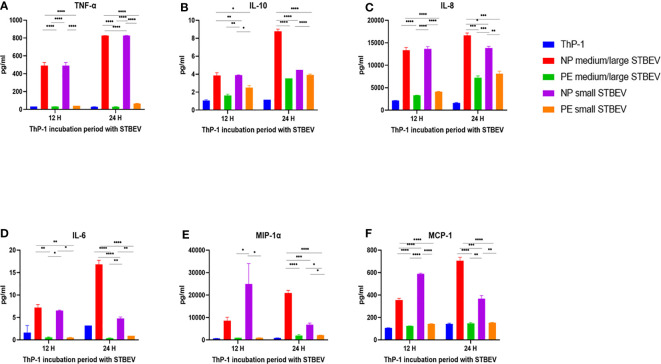
Multiplex array analysis of the supernatants of differentiated THP-1 cells treated with STBEVs from NP and PE placentae. Concentrations of TNF-α **(A)**, IL-10 **(B)**, IL-8 **(C)**, IL-6 **(D)**, MIP-α **(E)**, MCP-1 **(F)** released by THP-1 cells at 12 and 24 h incubation with STBEVs. Values calculated as Mean ± SEM of value from cells treated with 1 X 10^9^ of STBEVs. Bars without error bars represent data whose SEM are close to zero and could not be adequately plotted by prism. To determine significant difference in production, a one-way ANOVA was performed on data: *p≤ 0.05, **p≤ 0.01, ***p≤ 0.001 and ****p≤ 0.0001.

**Figure 10:**
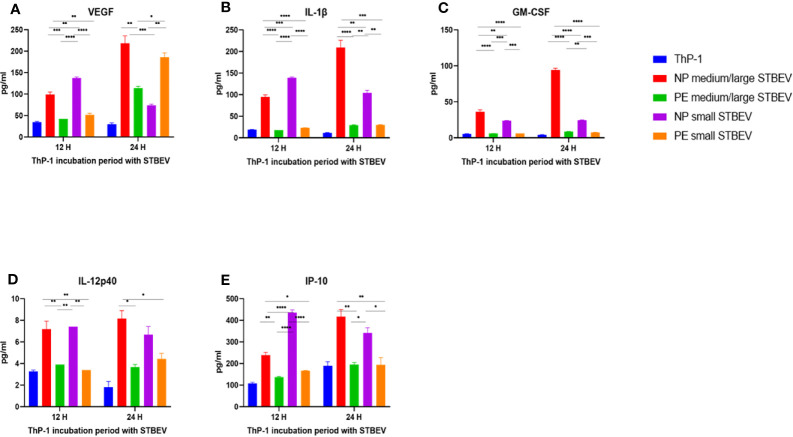
Multiplex array analysis of the supernatants of differentiated THP-1 cells treated with STBEVs from NP and PE placentae. Concentrations of VEGF **(A)**, IL-1β **(B)**, GM-CSF **(C)**, IL-12p40 **(D)** and IP-10 **(E)** released by THP-1 cells at 12 and 24 h incubation with STBEVs. Values calculated as Mean ± SEM of value from cells treated with 1 X 10^9^ of STBEVs. Bars without error bars represent data whose SEM are close to zero and could not be adequately plotted by prism. To determine significant difference in production, a one-way ANOVA was performed on data: *p≤ 0.05, **p≤ 0.01, ***p≤ 0.001 and ****p≤ 0.0001.

Multiplex array analysis was used to investigate the secreted levels of a range of cytokines, chemokines, growth factors, and soluble ligands following 12- and 24- h incubation of differentiated THP-1 cells with STBEV derived from NP and PE placentae (**Figures 9** and **10**). THP-1 cells challenged with NP medium/large STBEV appeared to show a predominantly pro-inflammatory profile, as evident from the significant higher quantities of TNF-α (**Figure 9A**), IL-6 (**Figure 9D**) IL-1β (**Figure 10B**), GM-CSF (**Figure 10C**) and IL-12p40 (**Figure 10D**), secreted in the supernatant compared to very feeble, if any, secretion by LOPE medium/large STBEV treated THP-1 cells at both time points (TNF-α, IL-1β, IL-6, GM-CSF = p<0.0001; IL-12p40 = p<0.001). Similarly, THP-1 cells challenged with NP small STBEV in comparison to LOPE small STBEV revealed the same trend as medium/large STBEV. NP medium/large STBEV treated THP-1 cells released around 2-fold higher levels of anti-inflammatory cytokine IL-10 (**Figure 9B**) compared to LOPE medium/large STBEV (p = 0.0026 at 12 hours, <0.0001 at 24 hours) at both time points. There was only a significant difference between NP small STBEV treated THP-1 release of IL-10 at 12 h in comparison to LOPE small STBEV (p=0.0204). Although IL-10 transcripts were upregulated in LOPE small STBEV, this is not consistent with the protein production by THP-1 cells after longer incubation with LOPE small STBEV.

Chemokines IL-8 (**Figure 9C**), MIP-1α (**Figure 9E**), MCP-1 (**Figure 9F**) and IP-10 (**Figure 10E**) were also secreted in higher quantities in response to NP medium/large STBEV compared to LOPE medium/large STBEV, as well as NP small STBEV compared to LOPE small STBEV. Particularly, NP medium/large STBEV released about 4 and 2.3 folds more IL-8 in treated THP-1 cells compared to LOPE medium/large STBEV (12 hours: p≤ 0.0001, 24 hours: p = 0.0001, **Figure 9C**) at 12 and 24 h, respectively. Similarly, IL-8 was secreted 3.3 and 1.7 fold more by THP-1 cells when induced with NP small STBEV compared to LOPE small STBEV (12 h: p≤ 0.0001, 24 h: p = 0.0014, **Figure 9C**). Both NP medium/large STBEV and NP small STBEV stimulated the IL-8 release of over 12,500 pg/mL, whilst LOPE medium/large STBEV and LOPE small STBEV also seemed to increase production of IL-8 but at a lower level (12 h: < 5,000 pg/mL, 24 h: > 5,000 pg/mL). Thus, NP STBEV challenged THP-1 cells secreted more IL-8 than LOPE STBEV.

In addition, NP medium/large STBEV produced 10.5 fold more MIP-1 α in THP-1 cells compared to LOPE medium/large STBEV at 24 h (p ≤ 0.0001, **Figure 9F**). NP small STBEV also induced 24.8 and 3.1 fold more MIP-1α than LOPE small STBEV (12 h: p≤ 0.0001, 24 h: p≤ 0.05, **Figure 9E**) at 12 and 24 h, respectively. Intriguingly, NP small STBEV produced 25 fold more MIP-1α after 12 h of incubation than NP medium/large STBEV (p≤ 0.0001); this effect was then reversed at 24 h though not significantly. Thus, our results revealed that MIP-1α was released in greater quantities by THP-1 cells when challenged with NP STBEV in comparison to cells challenged with LOPE STBEV.

Finally, at 12 h,VEGF was secreted in higher quantities by THP-1 challenged with NP medium/large STBEV (2.3 fold) and small STBEV (2.7 fold) compared to LOPE medium/large STBEV and small STBEV (p = 0.0006 (12 h) p<0.000.1 (24 h); **Figure 10A**); whilst at 24 h, THP-1 challenged with LOPE small STBEV secreted up to 2-fold more VEGF than NP small STBEV (p<0.05; **Figure 10A**).

Overall, our multiplex data revealed that medium/large and small STBEVs from LOPE placenta were less pro-inflammatory than those derived from NP placentas. These results largely corroborated the results of our gene expression analysis by RT-qPCR.

## Discussion

PE, most distinctly EOPE, is thought to originate from poor placentation in the first half of pregnancy, leading to ineffective remodelling of the uterine spiral arteries. This causes a high-pressure pulsatile flow in the intervillous space, which in turn, causes excess oxidative and hydrostatic stress to the placenta (1). Thus, EOPE has been associated with a vigorous maternal systemic inflammatory response to a damaged placenta (44), significant fetal growth restriction, and the increasing amount of STBEV shedding with different phenotype and cargo compared to a healthy pregnancy (37, 45). The late-onset disease is subject to several different theories of pathophysiology, two prominent ones being trophoblast villus overcrowding leading to oxidative stress and maladaptation of the maternal cardiovascular system as putative causes. Despite this, a role for the placenta is still evident since delivery brings about an end to PE-associated symptoms in both EOPE as well as LOPE. STBEV (medium/large and small STBEV) have been suggested to play different roles in the pathophysiology, with medium/large STBEV playing a more significant role in pro-inflammatory events compared to small STBEV (46). We decided to use the THP-1 cell line to avoid patient-related variations using PBMCs. By using PMA-differentiated THP-1 cells, we wanted to understand the likely true effect of STBEV on both circulating monocytes and tissue macrophages rather than just tissue macrophages. Therefore, we hypothesised that the LOPE placenta would release STBEVs, which might have an immunomodulatory impact similar to EOPE, i.e., pro-inflammatory. However, we observed a significantly weakened inflammatory effect of STBEVs isolated from LOPE placentae on THP-1 cells. While we could not find a perfect gestational age-matched control for the LOPE placentas, we ensured that the average difference between the two groups was not more than two weeks. In addition, all samples were collected within the last trimester. We used the same EV particle number to control EV variability that can be attributed to gestation age.

Our study shows that both STBEV types (medium/large STBEV and small STBEV) interact with macrophages and modulate their functions. Flow cytometry revealed co-localisation of medium/large STBEV derived from both NP and LOPE placentae to THP-1 macrophages. Confocal microscopy confirmed that this co-localisation was secondary to the internalisation of the STBEV. PLAP-based ELISA showing that THP-1 cells internalised placental-derived EV further confirmed this. THP-1 cells do not express PLAP, and the detection of PLAP in STBEV treated cells after washing suggests internalisation or/and membrane fusion of the STBEVs and THP-1 cells. This is in line with previous work showing mature PBMCs from NP donors phagocytosed SW71 trophoblast cell-derived EV (47) as well as medium/small STBEV isolated from dual perfusion (30). When assessing PLAP levels within the THP-1 cells, we were unable to see any significant difference in the uptake of NP and LOPE STBEV. We were able to determine enhanced uptake of medium/large STBEV compared to small STBEV in both NP and LOPE.

Analysis of gene expression *via* RT-qPCR and cytokine/chemokine secretion *via* multiplex array revealed that exposure of LOPE STBEV to THP-1 macrophages had significantly less inflammatory impact compared to NP STBEV. NP STBEV, especially NP medium/large STBEV, induced a pro-inflammatory response by THP-1 macrophages. Previous studies have shown that NP placental microvesicles induced significant levels of TNF-α, MIP-1α, IL-1α, IL-1β, IL-6 and IL-8 (30, 48) and an increased release of IFN-γ, IL-12, IL-18 and TNF-α by PBMCs (33). Similarly, NP medium/large STBEV derived from *in vitro* explant cultures from human placentae have been shown to enhance secretion of IL-1β, IL-6 and IL-8 by PBMCs (31). Correspondingly, exosomes derived from SW71 trophoblast cells have been shown to increase monocyte migration and production of IL-1β, IL-6, G-CSF, GM-CSF and TNF-α (47). Collectively, our NP results are consistent with these studies. The pro-inflammatory microenvironment and cytokines such as IL-1, IL-6, leukocyte inhibitory factor (LIF) (49), IFN-γ (50), IL-8, TNF-α, and MCP-1 are necessary for implantation, spiral artery remodelling, and parturition (51). TNF-α engineers local cytokine balance at the decidua that facilitates maternal-feto-placenta unit interaction (52). MCP-1 attracts macrophages into the endometrium to maintain M1/M2 balance and prevent rejection of the foetus earlier in pregnancy (53). TGF-β controls apoptosis and proliferation of endometrial cells and facilitates endometrial receptivity during the peri-implantation period (54). IL-1 β, IL-6, TNF-α and IL-8 expression is increased in the myometrium and cervix during parturition (55). IL-1β plays a role in initiating parturition by causing calcium influx into smooth muscle cells (56). IL-10 is increased in the placenta of normal pregnancy compared to high-risk pregnancies (57) and regulates trophoblastic invasion (58), regressing the pro-inflammatory microenvironment (59) that occurs in early pregnancy, and promoting angiogenesis due to the production of trophoblastic VEGF-C and aquaporin 1. Shortly before parturition, its level decline in order to facilitate the onset of labour (60). IL-6 activates and differentiates B and T cells (61), and stimulates M2 macrophage polarisation, promoting invasion and migration of macrophages (61). The individual functions of a number of these cytokines and their roles in implantation, pregnancy and parturition still require further research. Collectively, our NP results are consistent with these studies. Most surprising, however, was the significantly lower pro-inflammatory effect seen with LOPE STBEV. We made an effort, in three different ways, to confirm that the STBEV in both conditions were being internalised by the THP-1 cells; this ruled out the possibility that LOPE vesicles were not causing a pro-inflammatory effect because they were not being internalised.

Small STBEVs have not been extensively studied, but some groups have suggested that NP placenta explants derived exosomes have immunosuppressive roles (62, 63). Our study shows that small NP STBEVs are capable of causing an inflammatory phenotype while LOPE small STBEVs echo the results that we obtained from medium/large STBEV in that they did not display the expected pro-inflammatory response. Thus, in contrast to EOPE, any inflammation in LOPE is unlikely to be because of crosstalks between macrophages and STBEVs. Alternative inflammatory mechanisms may involve interactions with other immune cells in the decidua, such as the uterine NK cells or lymphocytes, or non-immune cells such as decidual stroma cells. Similarly, systemic inflammation may be propagated through STBEVs interaction with other cells in the circulation such as neutrophils or B-cells, or by directly activating the complement system that can stimulate the inflammatory response in LOPE (26). Tannetta et al. provided evidence that STBEV isolated from NP can cause platelet activation, which is augmented by PE STBEV (64). Increased platelet reactivity due to exposure to STBEV from PE might correlate with the increased thrombotic risk associated with PE, possibly leading to the inflammatory response (64). STBEV have also been shown to have anti-angiogenic and hypertensive effects, preventing endothelial cell monolayer growth *in vitro* as well as inhibiting relaxation of pre-constricted blood vessels *in vivo* (65–67).

Our study also differs from the observations of Abumaree et al. who noticed that placenta debris have an immunosuppressive effect on PBMCs. This might be because 1) pooled PBMCs were used with their inherent patient variability; 2) placenta debris was isolated from explants and not perfused placentas, and 3) the placenta debris obtained did not discriminate into the subtypes of EVs and may contain other materials beside EVs. Plasma EV derived from PE patients in comparison to those from NP have been shown to affect monocyte functions, such as altered phagocytosis-associated molecular pattern, decreased chemotactic and migratory activity, and increased adhesion (68). Unfortunately, this previous study did not focus on STBEV. Hence, we cannot conclude that the immunosuppressive effect exerted by the placenta debris is directly due to placenta-derived EV. Alternatively, STBEV derived from LOPE might have an impact. Although these studies have not separated microvesicles and exosomes and differentiated EOPE and LOPE, it is necessary to consider their findings when interpreting our results.

Studies have shown key differences between EOPE and LOPE, supporting the idea that these are separated disease entities. Women with PE show an imbalance between a pro-inflammatory and anti-inflammatory profile in CD4^+^ T-cell subsets with polarisation to T_H_17 profiles, predominantly in EOPE (69). Notably, this study reported endogenous plasma levels of IL-6, IL-7 and TNF-α to be significantly higher in the EOPE group than in the LOPE and control groups (69). Another study found substantially higher concentrations of Hsp70, TNF-α, IL-1β, IL-12, and sTNFRI in patients with EOPE compared to LOPE, as well as significantly lower IL-10 levels in the EOPE group (70). EOPE was, therefore, associated with higher maternal and foetal impairment, highlighting differences in the pathophysiology between EOPE and LOPE (70).

Previously, the effects of STBEV from LOPE placenta on immune function. Our data suggest that the differences seen in immune function have not been examined. in EOPE and LOPE may well in part be mediated by the differences in the effects of STBEV from these disease states. In addition, the apparent pro-inflammatory phenotype seen in LOPE might not be caused by a direct effect of STBEVs on macrophages but rather an effect on another yet to be determined immune or non-immune cell type(s) (e.g., uterine NK cells, T cells, neutrophils, platelets), or through direct activation of the complement system by the STBEVs.

## Data Availability Statement

The original contributions presented in the study are included in the article/supplementary material. Further inquiries can be directed to the corresponding author.

## Ethics Statement

The studies involving human participants were reviewed and approved by Oxford research ethics committee. The patients/participants provided their written informed consent to participate in this study.

## Author Contributions

UK, CR, and MV conceived the idea. TA, CM-M, WZ, LK, NK, KW, FA, DT, and EM performed the bench experiments. TA and CM-M analysed the data. TA and AC performed bioinformatics. TA, UK, CR, and MV wrote the first draft. All authors contributed to the article and approved the submitted version.

## Funding

This work was funded by Medical Research Council (MR/J003360/1).

## Conflict of Interest

The authors declare that the research was conducted in the absence of any commercial or financial relationships that could be construed as a potential conflict of interest.

The reviewer AK declared a shared affiliation, with no collaboration, with one of the authors UK to the handling editor at the time of the review.
